# Development and validation of a new set of indicators to assess the quality of maternal and child nutritional care at the primary care

**DOI:** 10.3389/fmed.2022.1011940

**Published:** 2022-12-07

**Authors:** Monica Ancira-Moreno, Isabel Omaña-Guzmán, Arturo Cuauhtémoc Bautista-Morales, Omar Acosta-Ruiz, Sonia Hernández Cordero, Soraya Burrola-Méndez, Mireya Vilar-Compte, Eric Monterrubio Flores, Martha Kaufer-Horwitz, Cecilia Pérez Navarro, Cinthya Muñoz-Manrique, Mónica Mazariegos, Alejandra Trejo-Domínguez, Belen Sánchez Muzquiz, Ariana Cajero, Mauro Brero, Matthias Sachse, Fernanda Cobo Armijo

**Affiliations:** ^1^Department of Health, Universidad Iberoamericana, Mexico City, Mexico; ^2^Centro de Investigación en Evaluación y Encuestas, Instituto Nacional de Salud Pública, Cuernavaca, Mexico; ^3^Instituto de Investigaciones para el Desarrollo con Equidad (EQUIDE), Universidad Iberoamericana, Mexico City, Mexico; ^4^Department of Public Health, Montclair State University, Montclair, NJ, United States; ^5^Centro de Investigación en Nutrición y Salud, Instituto Nacional de Salud Pública, Cuernavaca, Mexico; ^6^Clínica de Obesidad y Trastornos de la Conducta Alimentaria, Departamento de Endocrinología y Metabolismo, Instituto Nacional de Ciencias Médicas y Nutrición Salvador Zubirán, Mexico City, Mexico; ^7^Departamento de Nutrición y Bioprogramación, Instituto Nacional de Perinatología, Mexico City, Mexico; ^8^INCAP Research Center for the Prevention of Chronic Diseases (CIIPEC), Institute of Nutrition of Central America and Panama (INCAP), Guatemala City, Guatemala; ^9^United Nations International Children’s Emergency Fund (UNICEF), Mexico City, Mexico

**Keywords:** quality of health care, quality indicators, health care, maternal malnutrition, child malnutrition, primary health care, nutritional care, quality of nutritional care

## Abstract

**Introduction:**

Maternal and child malnutrition is a worldwide public health problem with short, medium, and long-term adverse consequences for both mother and child. In Mexico, maternal and child malnutrition represents a serious public health problem that must be urgently addressed. In this context, Primary Health Care (PHC) plays an important role in the prevention, detection, monitoring, and treatment of the different forms of maternal and child malnutrition. Assessing the quality of nutritional care offered at this level of care is necessary in order to improve it; however, there are no indicators for the evaluation of this quality. Therefore, this study aimed at developing a set of indicators to assess the quality of maternal and child nutritional care at PHC.

**Methods:**

We developed indicators for different stages of life: preconception, pregnancy, infancy, and preschool age. A systematic review of the literature on clinical guidelines for the prevention, diagnosis, and treatment of the different forms of malnutrition was carried out; the recommendations of the guidelines evaluated with good quality were extracted.

**Results:**

Based on these recommendations, 22 indicators were constructed. A pilot study was carried out to validate the indicators and 16 indicators were selected to assess the maternal and child nutritional care at PHC.

## Introduction

Maternal and child malnutrition remains a global health problem. The coexistence of undernutrition and obesity increases not only the risk of maternal morbidity and mortality but also impacts the fetal growth and development with short and long-term consequences (1). As reported by the Global Burden of Disease Study 2019, maternal and child malnutrition (including low birth weight, short gestation, child growth failure, suboptimal breastfeeding, and low intake of micronutrients), represents one of the two leading level 2 risk factors for the lose of disability-adjusted life years (2).

In Mexico, based on the 2020 National Survey of Health and Nutrition about COVID-19 (3), 76% of women of reproductive age (20–49 years) had excess weight (overweight/obesity). Both conditions increase the risk of pregnancy-related complications and adverse long-term consequences for both the offspring and the mother (4). Regarding malnutrition in children under 5 years of age, 13.9% had stunting while the national prevalence of overweight and obesity was 8.4%; both prevalences were higher in rural areas (18.5 and 8.5%, respectively). In addition, only 28% of infants received exclusive breastfeeding for 6 months (3). Improving women’s health and nutritional status through prenatal care during preconception, pregnancy and postpartum is critical for ensuring positive short and long-term outcomes for both, the mother and child (5).

Primary health care (PHC) aims to ensure the highest possible level of health and wellbeing of an individual and populations; it ranges from health promotion, prevention, treatment, rehabilitation, and palliative care (6). It has been shown that PHC is the most efficient and effective strategy to improve the health of populations, and nutrition care is a critical component of PHC (6, 7). To address malnutrition in all its forms, a life-course approach is necessary and should be a central part of PHC (7). Therefore, PHC is essential to reduce and prevent all forms of maternal and child malnutrition and the nutritional care offered at this level must be of high quality (8).

Currently, there are essential nutritional actions addressed to eradicate maternal and child malnutrition in all its forms. Despite the proven knowledge, most countries have failed to reach their targets, sometimes due to the lack of coverage but also for neglecting specific goals. The nutritional interventions that are cost-effective and group-focused must be prioritized, considering the social context and the economic resources of each entity. In addition, for the improvement of the quality of care, it is necessary to reduce the gap between the scientific evidence, the politics and governance, and the resources destined to the centers of primary care (9–11).

According to the World Health Organization (WHO), the quality of care is defined as the degree to which health services for individuals and populations increase the likelihood of desired health outcomes, and this must be according to the best evidence-based professional knowledge (12). In the field of nutrition, there is no consensus on defining the standards and indicators to evaluate the quality and performance of nutritional care (13, 14). Some researchers have evaluated the quality of nutritional care at PHC using a conceptual framework developed to assess the quality of care in general (15).

Currently, there is limited information about the description and development of indicators for quality of nutritional care during preconception, pregnancy, postpartum, and infancy. In Mexico, all the efforts are focused on quality of care to identify obstetric risk factors without regard to nutritional care (16). It is important to provide support to governments and decision makers to integrate high-quality nutrition care into PHC and improve care for achieving interventions addressing maternal and child malnutrition.

Therefore, we aimed to develop and validate quality of care indicators for maternal and child nutritional care on PHC in the environment of the Mexican Health System. Having indicators that allow us to evaluate the quality of nutritional care in the aforementioned stages of life will allow us to identify improvement areas in the quality of care offered in PHC in Mexico.

## Materials and methods

The development of the indicators to evaluate the quality of maternal and child nutritional care at PHC was carried out by following six steps. In Supplementary Figure 1 is shown the flowchart of the whole process of the development of the indicators.

### Step 1. Systematic literature search

A systematic literature search was carried out in PubMed and websites for clinical practice guidelines, guidelines and position statements related to the prevention, diagnosis, and management of all forms of malnutrition during preconception, pregnancy, postpartum, and infancy and preschool children. To assess the quality of these documents, the methodology of the Appraisal Of Guidelines For Research and Evaluation II (AGREE II) (17) instrument was used. The search algorithms and limits for each life stage are presented in Supplementary Table 1. Subsequently, the recommendations for a specific stage of life related to the prevention, diagnosis, and management of malnutrition from the guidelines and documents with “acceptable quality” according to AGREE II instrument (17) were extracted.

### Step 2. Construction of indicators to measure the quality of maternal and child nutritional care

The recommendations, based on the results of the systematic literature search, were reviewed by two researchers with experience in medical care at PHC. Recommendations whose measurement required information sources other than those existing in health units and those that involved care at a higher level than PHC were eliminated.

In order to reduce the number of recommendations while maintaining those with the highest degree of recommendation and measurement feasibility, a prioritization matrix was constructed. For this, we used the scoring system according to compliance with criteria based on the degree of recommendation, the feasibility of application, and ease of obtaining the sample necessary for measurement and relevance (Table 1).

**TABLE 1:** Scoring system used to build the recommendation prioritization matrix.

A. Grade of recommendation	Points
- Grade of recommendation A, B, strong recommendation - Grade of recommendation C, D, regular recommendation - Grade of recommendation E, good practice point, weak recommendation - The degree of recommendation is not specified	3 2 1 0
B. Feasibility of application (the score was assigned based on the sum of the following criteria)	Points
- Non-specialized knowledge - Specific moment of evolution - Specific recommendation	1 1 1
C. Sample necessary for the measurement, the score was assigned as follows:	Points
- There is a census - A subsample can be easily identified based on existing records, that is, the target groups have been identified in a card holder or similar record - A complex subsample can be identified based on existing records, but a subgroup is required to be identified from a card rack or similar source - There is no specific record in the unit	3 2 1 0
D. Relevance, the score was assigned based on the sum of the following criteria:	Points
- Frequency of the event - Involves complications or death if the activity is not performed - High degree of recommendation	1 1 1

The review was conducted in pairs by experts in quality of care and the disagreements were resolved through discussion and consensus. The values of each criterion were added to obtain a total score ranging from 0 to 12 points, considering the recommendation to be more useful when the score was higher. The highest scores were selected for each step of the care process (prevention, diagnosis, treatment, and follow-up) at each stage (preconception, pregnancy, postpartum, infancy, and preschool age).

The selected recommendations were reviewed by a group of experts in nutrition and quality of care to reduce the set to a manageable number for collecting information in the field and to be valid for the context of care in health services in Mexico.

### Step 3. Technical sheet of quality indicators

For the development of the indicators, the methodology suggested by Saturno-Hernández (18) was followed, and the components of the indicators were reflected in standardized technical sheets (19) developed by the same author.

### Step 4. Data collection instruments

Based on the structure of the indicators and their formulas, a data collection instrument was designed using Excel spreadsheets to facilitate its use in any electronic device.

### Step 5. Pilot study

A pilot study was carried out to evaluate the reliability of the 22 indicators and the feasibility of their application. This study was performed in five PHC rural and urban units belonging to the Mexican Ministry of Health in the State of Mexico.

The clinical records and nutritional control cards were the inputs to obtain the necessary information to evaluate the indicators. Each indicator was evaluated by two independent researchers by reviewing a systematic random sample of 30 clinical records and nutritional control cards for each life stage (18).

The sampling was carried out through patient records on the Child Nutrition Status Control cards (SINBA-SIS-18-P) and Comprehensive Care for Pregnancy, Puerperium, and Lactation Period (SINBA-SIS-38-P) (19) from 2020 and 2021.

### Step 6. Analysis and selection of indicators

The reliability of the indicators was evaluated by inter-rater agreement based on the results of the kappa adjusted for prevalence and inter-rater bias (PABAK). Indicators with a result equal to or greater than 0.4 were considered reliable, taking into account the Benchmark scale (20).

Feasibility was established based on the possibility of being measured through the available data sources in the medical units, recording the difficulties that arose during measurement.

Based on the reliability and feasibility results, the group of experts carried out a second prioritization exercise to keep a feasible number of indicators that would be manageable by health personnel.

## Results

### Selected recommendations

Of the 922 recommendations identified in this work, 124 were excluded (7 in preconception, 20 in pregnancy, 32 in postpartum, 42 in infancy, and 23 in preschool) since they were related to the second level of care or required information other than the available in health units. With the selected recommendations, 22 composite indicators were constructed and tested in the pilot study. Table 2 shows the 22 guideline-based quality indicators of maternal and child nutritional care at primary care with their main characteristics (name, type of indicator, description, and formula). The selected indicators were all process indicators since, as Donabedian (21) points out, this approach denotes what is really done in health care.

**TABLE 2:** Twenty two guideline-based indicators to assess the quality of maternal and child nutritional care at primary health care.

Life stage	Name of indicator	Type of indicator	Description	Formula
	Weight control strategies	Process	Percentage of patients who have been prescribed dietary, exercise, medical or surgical strategies for weight management 3 months before their pregnancy	Number⁢of⁢patients⁢during⁢the⁢preconception⁢period⁢who⁢havebeen⁢prescribed⁢dietary,exercise,medical,or⁢surgical⁢strategies⁢forweight⁢management⁢three⁢months⁢before⁢their⁢pregnancyTotal⁢number⁢of⁢patients⁢during⁢the⁢preconception⁢period⁢x⁢ 100
	
Preconception	Anthropometric assessment	Process	Percentage of patients with complete anthropometric assessment (Height, weight and Body Mass Index)	Number⁢of⁢patients⁢in⁢the⁢preconception⁢stage⁢who⁢underwent⁢acomplete⁢anthropometric⁢evaluationTotal⁢number⁢of⁢patients⁢during⁢the⁢preconception⁢period⁢x⁢ 100
	
	Folic acid supplementation	Process	Percentage of patients who are prescribed folic acid three months before their pregnancy	$\frac{\begin{array}{c} \mathrm{Number}\,\mathrm{of}\,\mathrm{patients}\,\mathrm{in}\,\mathrm{the}\,\mathrm{preconception}\,\mathrm{stage}\,\mathrm{who}\,\mathrm{were}\,\mathrm{prescribed}\\ \mathrm{folic}\,\mathrm{acid}\,\mathrm{supplementation}\,\mathrm{in}\,\mathrm{the}\,\mathrm{three}\,\mathrm{months}\,\mathrm{prior}\,\mathrm{to}\,\mathrm{their}\,\mathrm{pregnancy} \end{array}}{\mathrm{Total}\,\mathrm{number}\,\mathrm{of}\,\mathrm{patients}\,\mathrm{during}\,\mathrm{the}\,\mathrm{preconception}\,\mathrm{period}}\,\mathrm{x100}$

	Glycemic control during pregnancy	Process	Percentage of patients with normal values in the oral glucose tolerance curve during pregnancy	$\frac{\begin{array}{c} \mathrm{Number}\,\mathrm{of}\,\mathrm{pregnant}\,\mathrm{patients}\,\mathrm{with}\,\mathrm{normal}\,\mathrm{values}\,\mathrm{in}\,\mathrm{the}\,\mathrm{oral}\,\mathrm{glucose}\\ \mathrm{tolerance}\,\mathrm{curve} \end{array}}{\mathrm{Total}\,\mathrm{number}\,\mathrm{of}\,\mathrm{pregnant}\,\mathrm{patients}}\,\mathrm{x100}$
	
	Supplementation in pregnancy	Process	Percentage of patients with folic acid supplementation during the first trimester of pregnancy	$\frac{\begin{array}{c} \mathrm{Number}\,\mathrm{of}\,\mathrm{pregnant}\,\mathrm{patients}\,\mathrm{who}\,\mathrm{were}\,\mathrm{prescribed}\,\mathrm{folic}\\ \mathrm{acid}\,\mathrm{supplementation}\,\mathrm{during}\,\mathrm{the}\,\mathrm{first}\,\mathrm{trimester}\,\mathrm{of}\,\mathrm{pregnancy} \end{array}}{\mathrm{Total}\,\mathrm{number}\,\mathrm{of}\,\mathrm{pregnant}\,\mathrm{patients}}\,\mathrm{x100}$
	
	Anemia screening	Process	Percentage of pregnant patients who have been screened for anemia	Number⁢of⁢pregnant⁢patients⁢to⁢whom⁢screening⁢for⁢anemia⁢wasprovided⁢during⁢the⁢first⁢pregnancy⁢consultation⁢and⁢around⁢ 28gestational⁢weeksTotal⁢number⁢of⁢pregnant⁢patients⁢x100
	
Pregnancy	Preeclampsia screening	Process	Percentage of pregnant patients with blood pressure measurement and urinalysis	$\frac{\begin{array}{c} \mathrm{Number}\,\mathrm{of}\,\mathrm{pregnant}\,\mathrm{patients}\,\mathrm{who}\,\mathrm{underwent}\,\mathrm{blood}\,\mathrm{pressure}\\ \mathrm{measurement}\,\mathrm{and}\,\mathrm{urinalysis}\,\mathrm{at}\,\mathrm{each}\,\mathrm{medical}\,\mathrm{consultation} \end{array}}{\mathrm{Total}\,\mathrm{number}\,\mathrm{of}\,\mathrm{pregnant}\,\mathrm{patients}}\,\mathrm{x100}$
	
	Screening for risk factors for preeclampsia	Process	Percentage of patients with identified risk factors for preeclampsia	$\frac{\begin{array}{c} \mathrm{Number}\,\mathrm{of}\,\mathrm{pregnant}\,\mathrm{patients}\,\mathrm{who}\,\mathrm{were}\,\mathrm{screened}\,\mathrm{for}\,\mathrm{risk}\,\mathrm{factors}\\ \mathrm{for}\,\mathrm{preeclampsia}\,\mathrm{at}\,\mathrm{the}\,\mathrm{first}\,\mathrm{consultation} \end{array}}{\mathrm{Total}\,\mathrm{number}\,\mathrm{of}\,\mathrm{pregnant}\,\mathrm{patients}}\,\mathrm{x100}$
	
	Adequate follow-up	Process	Percentage of patients with adequate prenatal follow-up	$\frac{\mathrm{Number}\,\mathrm{of}\,\mathrm{patients}\,\mathrm{with}\,\mathrm{adequate}\,\mathrm{prenatal}\,\mathrm{follow}-\text{up}}{\mathrm{Total}\,\mathrm{number}\,\mathrm{of}\,\mathrm{pregnant}\,\mathrm{patients}}\,\mathrm{x100}$
	
	Nutritional evaluation and vitamin supplementation in adolescent pregnancy	Process	Percentage of adolescent pregnant patients with nutritional evaluation and adequate vitamin supplementation	$\frac{\begin{array}{c} \mathrm{Number}\,\mathrm{of}\,\mathrm{adolescent}\,\mathrm{pregnant}\,\mathrm{patients}\,\mathrm{with}\,\mathrm{nutritional}\,\mathrm{evaluation}\,\mathrm{and}\\ \mathrm{adequate}\,\mathrm{vitamin}\,\mathrm{supplementation} \end{array}}{\mathrm{Total}\mathrm{number}\mathrm{of}\mathrm{adolescent}\mathrm{pregnant}\mathrm{patients}.}\,\mathrm{x100}$
	
Postpartum	Guidance on techniques for effective latching, breast massage and milk expression	Process	Percentage of patients during the breastfeeding period who had guidance on techniques for effective latching, breast massage, and milk expression	$\frac{\begin{array}{c} \mathrm{Number}\,\mathrm{of}\,\mathrm{patients}\,\mathrm{during}\,\mathrm{the}\,\mathrm{breastfeeding}\,\mathrm{period}\,\mathrm{with}\\ \mathrm{guidance}\,\mathrm{on}\,\mathrm{techniques}\,\mathrm{for}\,\mathrm{effective}\,\mathrm{latching},\mathrm{breast}\,\mathrm{massage},\\ \mathrm{and}\,\mathrm{milk}\,\mathrm{expression} \end{array}}{\mathrm{Total}\mathrm{number}\mathrm{of}\mathrm{patients}\mathrm{during}\mathrm{the}\mathrm{breastfeeding}\mathrm{period}.}\,\mathrm{x100}$
	
	Guidance on postpartum weight control	Process	Percentage of patients during the postpartum period with counseling about weight control	$\frac{\begin{array}{c} \mathrm{Number}\,\mathrm{of}\,\mathrm{patients}\,\mathrm{during}\,\mathrm{the}\,\mathrm{postpartum}\,\mathrm{period}\,\mathrm{who}\,\mathrm{received}\\ \mathrm{counseling}\,\mathrm{about}\,\mathrm{weight}\,\mathrm{control} \end{array}}{\mathrm{Total}\mathrm{number}\mathrm{of}\mathrm{patients}\mathrm{during}\mathrm{the}\mathrm{postpartum}\mathrm{period}.}\,\mathrm{x100}$
	
	Nutritional and physical activity recommendations.	Process	Percentage of patients with obesity and nutritional and physical activity recommendations in the postpartum period	$\frac{\begin{array}{c} \mathrm{Number}\,\mathrm{of}\,\mathrm{postpartum}\,\mathrm{patients}\,\mathrm{with}\,\mathrm{obesity}\,\mathrm{who}\,\mathrm{were}\,\mathrm{provided}\\ \mathrm{with}\,\mathrm{nutritional}\,\mathrm{and}\,\mathrm{physical}\,\mathrm{activity}\,\mathrm{recommendations} \end{array}}{\mathrm{Number}\,\mathrm{of}\,\mathrm{postpartum}\,\mathrm{patients}\,\mathrm{with}\,\mathrm{obesity}}\,\mathrm{x100}$

	Promotion of exclusive breastfeeding, continued breastfeeding and complementary feeding	Process	Percentage of patients whose mothers had promotion of exclusive breastfeeding, continued breastfeeding, and complementary feeding	$\frac{\begin{array}{c} \mathrm{Number}\,\mathrm{of}\,\mathrm{patients}\,\mathrm{under}\,\mathrm{two}\,\mathrm{years}\,\mathrm{old}\,\mathrm{whose}\,\mathrm{mothers}\\ \mathrm{had}\,\mathrm{promotion}\,\mathrm{of}\,\mathrm{exclusive}\,\mathrm{breastfeeding},\mathrm{continued}\,\mathrm{breastfeeding},\\ \mathrm{and}\,\mathrm{complementary}\,\mathrm{feeding} \end{array}}{\mathrm{Total}\,\mathrm{number}\,\mathrm{of}\,\mathrm{patients}\,\mathrm{under}\,\mathrm{two}\,\mathrm{years}\,\mathrm{old}}\,\mathrm{x100}$
	
	Assessment of nutritional status	Process	Percentage of infants who had an assessment of nutritional status	$\frac{\mathrm{Number}\,\mathrm{of}\,\mathrm{infants}\,\mathrm{with}\,\mathrm{an}\,\mathrm{assessment}\,\mathrm{of}\,\mathrm{nutritional}\,\mathrm{status}}{\mathrm{Total}\,\mathrm{number}\,\mathrm{of}\,\mathrm{infants}}\,\mathrm{x100}$
	
Infancy	Recommendation to reduce energy intake and fast food in infants with obesity	Process	Percentage of patients under 2 years old with obesity who have received recommendations to reduce energy and fast-food intake	$\frac{\begin{array}{c} \mathrm{Number}\,\mathrm{of}\,\mathrm{infants}\,\mathrm{with}\,\mathrm{obesity}\,\mathrm{who}\,\mathrm{recieved}\,\mathrm{recommendations}\\ \mathrm{to}\,\mathrm{reduce}\,\mathrm{energy}\,\mathrm{and}\,\mathrm{fast}-\mathrm{food}\,\mathrm{intake} \end{array}}{\mathrm{Total}\,\mathrm{number}\,\mathrm{of}\,\mathrm{patients}\,\mathrm{under}\,\mathrm{two}\,\mathrm{years}\,\mathrm{with}\,\mathrm{obesity}}\,\mathrm{x100}$
	
	Follow-up of patients with malnutrition	Process	Percentage of patients with malnutrition who had adequate follow-up	$\frac{\begin{array}{c} \mathrm{Number}\,\mathrm{of}\,\mathrm{patients}\,\mathrm{under}\,\mathrm{two}\,\mathrm{years}\,\mathrm{old}\,\mathrm{with}\,\mathrm{malnutrition}\,\mathrm{who}\\ \mathrm{received}\,\mathrm{adequate}\,\mathrm{follow}-\mathrm{up} \end{array}}{\mathrm{Total}\,\mathrm{number}\,\mathrm{of}\,\mathrm{patients}\,\mathrm{under}\,\mathrm{two}\,\mathrm{years}\,\mathrm{with}\,\mathrm{malnutrition}}\,\mathrm{x100}$
	
	Timely detection and identification of risk factors for the development of iron deficiency anemia in patients under 2 years of age with malnutrition	Process	Percentage of patients under 2 years old with malnutrition who had timely detection and identification of risk factors for the development of iron deficiency anemia	$\frac{\begin{array}{c} \mathrm{Number}\,\mathrm{of}\,\mathrm{patients}\,\mathrm{under}\,\mathrm{two}\,\mathrm{years}\,\mathrm{old}\,\mathrm{with}\,\mathrm{malnutrition}\,\mathrm{who}\,\mathrm{had}\,\mathrm{timely}\\ \mathrm{detection}\,\mathrm{and}\,\mathrm{identification}\,\mathrm{of}\,\mathrm{risk}\,\mathrm{factors}\,\mathrm{for}\,\mathrm{the}\,\mathrm{development}\\ \mathrm{of}\,\mathrm{iron}\,\mathrm{deficiency}\,\mathrm{anemia} \end{array}}{\mathrm{Total}\,\mathrm{number}\,\mathrm{of}\,\mathrm{patients}\,\mathrm{under}\,\mathrm{two}\,\mathrm{years}\,\mathrm{with}\,\mathrm{malnutrition}}\,\mathrm{x100}$

	Physical activity and nutritional recommendations	Process	Percentage of patients in the range from 2 to 5 years old who received physical activity and nutritional recommendations	Number⁢of⁢patients⁢in⁢the⁢range⁢from⁢ 2⁢to⁢ 5⁢years⁢old⁢who⁢received⁢physicalactivity⁢and⁢nutritional⁢recommendationsTotal⁢number⁢of⁢patients⁢in⁢the⁢range⁢from⁢ 2⁢to⁢ 5⁢years⁢old⁢x100
	
	Preschool children with anthropometric assessment	Process	Percentage of patients in the range from 2 to 5 years old who had an anthropometric assessment on each consultation	Number⁢of⁢patients⁢in⁢the⁢range⁢from⁢ 2⁢to⁢ 5⁢years⁢old⁢who⁢had⁢ananthropometric⁢assessment⁢on⁢each⁢consultation.Total⁢number⁢of⁢patients⁢in⁢the⁢range⁢from⁢ 2⁢to⁢ 5⁢years⁢old⁢x100
	
Preschool age	Identification of risk factors for malnutrition in preschool children	Process	Percentage of patients in the range from 2 to 5 years old in whom risk factors for malnutrition are identified	Number⁢of⁢patients⁢in⁢the⁢range⁢from⁢ 2⁢to⁢ 5⁢years⁢oldin⁢whom⁢risk⁢factors⁢for⁢malnutrition⁢are⁢identifiedTotal⁢number⁢of⁢patients⁢in⁢the⁢range⁢from⁢ 2⁢to⁢ 5⁢years⁢old⁢x100
	
	Recommendation to reduce energy intake and fast food in preschool children with obesity	Process	Percentage of patients in the range from 2 to 5 years old with obesity who received recommendations to reduce total energy intake and fast food consumption	Number⁢of⁢patients⁢in⁢the⁢range⁢from⁢ 2⁢to⁢ 5⁢years⁢old⁢with⁢obesity⁢who⁢receivedrecommendations⁢to⁢reduce⁢total⁢energy⁢intake⁢and⁢fast⁢food⁢consumptionTotal⁢number⁢of⁢patients⁢in⁢the⁢range⁢from⁢ 2⁢to⁢ 5⁢years⁢old⁢with⁢obesity⁢x100

### Selected indicators

The reliability of the indicators obtained in the pilot study is shown in Table 3. For the preconception, no information was found in any of the five health centers evaluated in the pilot study; therefore, reliability could not be assessed. Similarly, no information was found on the following indicators: “Recommendation to reduce energy intake and fast food in infants with obesity,” “Follow-up of patients with malnutrition,” “Timely detection of risk factors for the development of iron deficiency anemia in patients under 2 years of age with malnutrition,” “Identification of risk factors for malnutrition in preschool children,” “Recommendation to reduce energy intake and fast food in preschool children with obesity,” and reliability could not be assessed.

**TABLE 3:** Reliability of the quality indicators of maternal and child nutritional care at primary care.

Name of the indicator and sub-indicator	PABAK (CI 95%)	Number of clinical records	Percentage compliance** (CI 95%)	
			Evaluator 1	Evaluator 2
**Weight control strategies**	–	0	–	–
Dietary recommendations	–	0	–	–
Physical activity/exercise recommendations	–	0	–	–
Pharmacological treatment recommendations	–	0	–	–
Surgical recommendations	–	0	–	–
**Anthropometric evaluation**	–	0	–	–
**Folic acid supplementation**	–	0	–	–
**Glycemic control in pregnancy**	1* (1.00–1.00)	33	0.0 (0.0–10.6)	0.0 (0.0–10.6)
**Supplementation in pregnancy**	0.87* (0.72–1.00)	46	8.7 (3.2–21.7)	6.5 (2.0–19.1)
Folic acid	0.90 (0.79–1.00)	46	65.2 (50.0–77.9)	69.6 (54.3–81.4)
Vitamin D	0.87 (0.76–0.99)	46	8.7 (3.2–21.7)	6.5 (2.0–19.1)
**Anemia screening**	0.60* (0.46–0.80)	44	29.5 (17.6–45.1)	22.7 (12.4–38.0)
Blood biometry request in the first consultation	0.64 (0.49–0.78)	44	75.0 (59.6–85.9)	65.9 (50.3–78.7)
Blood count request around 28 week of pregnancy	0.80 (0.64–0.99)	44	36.4 (23.2–52.0)	31.8 (19.4–47.5)
**Preeclampsia screening**	0.60* (0.41–0.75)	36	30.6 (17.3–48.1)	33.3 (19.4–50.9)
Blood pressure measurement	0.83 (0.64–1.00)	36	91.7 (76.1–97.4)	100.0 (90.3–100.0)
Application of urolabstix or general urine test	0.72 (0.48–0.95)	36	36.1 (21.7–53.6)	33.3 (19.4–50.9)
**Screening for risk factors for preeclampsia**	1* (1.00–1.00)	26	3.8 (0.5–25.4)	3.8 (0.5–25.4)
**Adequate follow-up**	0.65* (0.43–0.86)	51	33.3 (21.5–47.7)	23.5 (13.6–37.5)
Adequate number of medical consultations	0.65 (0.43–0.86)	51	33.3 (21.5–47.7)	23.5 (13.6–37.5)
Recorded the patient’s weight at each visit	0.92 (0.81–1.00)	51	98.0 (86.6–99.7)	98.0 (86.6–99.7)
**Nutritional evaluation and vitamin supplementation in adolescent pregnancy**	0.54* (0.18–1.00)	13	15.4 (3.1–51.0)	7.7 (0.8–46.9)
Nutritional diagnosis	0.54 (0.18–1.00)	13	38.5 (14.6–69.5)	30.8 (10.2–63.5)
Folic acid and iron prescription	0.84 (0.51–1.00)	13	30.8 (10.2–63.5)	38.5 (14.6–69.5)
**Guidance on techniques for effective latching, breast massage and milk expression**	1* (1.00–1.00)	9	0.0 (0.0–33.6)	0.0 (0.0–33.6)
**Guidance on postpartum weight control**	0.56* (0.12–1.00)	9	33.3 (8.1–73.8)	11.1 (0.9–62.9)
**Nutritional and physical activity recommendations**	1* (1.00–1.00)	8	0.0 (0.0–36.9)	0.0 (0.0–36.9)
Nutritional recommendations	0.75 (0.16–1.00)	8	25.0 (4.1–72.4)	12.5 (0.9–68.1)
Physical activity recommendations	1 (1.00–1.00)	8	0.0 (0.0–36.9)	0.0 (0.0–36.9)
**Promotion of exclusive breastfeeding, continued breastfeeding and complementary feeding**	0.83* (0.65–1.00)	24	8.3 (1.9–30.2)	16.67 (5.9–38.9)
Promotion of exclusive breastfeeding in children under 6 months	0.67 (0.44–0.99)	24	41.67 (22.9–63.1)	50.0 (29.7–70.3)
Promotion of continued breastfeeding after 6 months and up to 2 years	0.92 (0.74–1.00)	24	29.2 (13.7–51.5)	33.3 (16.7–55.5)
Complementary feeding recommendation from 6 months of age	0.75 (0.56–1.00)	24	58.3 (36.9–77.1)	45.8 (26.3–66.8)
**Assessment of nutritional status**	0.86* (0.65–1.00)	28	7.1 (1.6–26.3)	7.1 (1.6–26.3)
Dietary history	0.64 (0.41–0.94)	28	28.6 (14.3–48.9)	25.0 (11.8–45.3)
Social and economic history	0.79 (0.54–1.00)	28	42.9 (25.2–62.5)	39.3 (22.4–59.2)
Anthropometric measurements	0.78 (0.54–1.00)	28	67.9 (47.5–83.1)	64.3 (44.1–80.4)
Nutritional diagnosis	0.57 (0.25–0.89)	28	67.9 (47.5–83.1)	67.9 (47.5–83.1)
**Recommendation to reduce energy intake and fast food in infants with obesity.**	–	0	–	–
**Follow-up of patients with malnutrition**	–	0	–	–
**Timely detection and identification of risk factors for the development of iron deficiency anemia in patients under 2 years of age with malnutrition**	–	0	–	–
**Physical activity and nutritional recommendations**	1* (1.00–1.00)	6	0.0 (0.0–45.9)	0.0 (0.0–45.9)
Physical Activity/Exercise recommendations	1 (1.00–1.00)	6	0.0 (0.0–45.9)	0.0 (0.0–45.9)
Nutritional recommendations	0.67 (0.19–1.00)	6	16.7 (0.9–81.4)	0.0 (0.0–45.9)
**Preschool children with anthropometric assessment**	0.67* (0.19–1.00)	6	16.7 (0.9–81.4)	0.0 (0.0–45.9)
**Identification of risk factors for malnutrition in preschool children**	–	0	–	–
**Recommendation to reduce energy intake and fast food in preschool children with obesity**	–	0	–	–

PABAK, kappa adjusted for prevalence and inter-rater bias. The indicators are in bold letters and the sub-indicators that compose them are in normal letters. *Indicator is reliable. **Percentage of compliance with the indicators found by each evaluator in the review of clinical records.

Considering the aspects mentioned in “Materials and methods” section, we selected 16 indicators to assess the quality of nutritional maternal and child care at health units at PHC (Table 4). We decided to include indicators from the preconception stage (although no information was obtained) since it is a critical period that represents an opportunity to implement interventions aimed at improving the course of pregnancy and its results for the mother-child binomial (20).

**TABLE 4:** Final selected indicators to assess the quality of maternal and child nutritional care.

Life stage	Indicator name
Preconception	Weight control strategies
	Folic acid supplementation
Pregnancy	Supplementation in pregnancy
	Anemia screening
	Adequate follow-up
	Nutritional evaluation and vitamin supplementation in adolescent pregnancy
Postpartum	Guidance on techniques for effective latching, breast massage and milk expression
	Guidance on postpartum weight control
Infancy	Promotion of exclusive breastfeeding, continued breastfeeding and complementary feeding
	Assessment of nutritional status
	Recommendation to reduce energy intake and fast food in infants with obesity
	Follow-up of patients with malnutrition
	Timely detection and identification of risk factors for iron deficiency anemia in patients under 2 years of age with malnutrition
Preschool age	Physical activity and nutritional recommendations
	Preschool children with anthropometric assessment
	Recommendation to reduce energy intake and fast food in preschool children with obesity

## Discussion

We carried out, for the first time in Mexico, a systematic and validated process for the development of indicators to assess the quality of maternal and child nutritional care at PHC that can be implemented within the Mexican health system.

The activities evaluated by the 16 selected indicators coincide with the recommended evidence-based nutrition actions over the life course by WHO (9) for improving maternal and child nutrition. The proposed indicators have the potential to identify areas of improvement in the process of nutritional care at PHC since they include the evaluation of actions related to the prevention and promotion, diagnosis, and treatment of the different forms of malnutrition in the period of preconception, pregnancy, postpartum, infancy, and preschool stage. Furthermore, the developed indicators incorporate key processes recommended in the construction of quality of care indicators (22, 23). In addition, it should be noted that their development is based on a rigorous methodology that includes the selection of recommendations with a high degree of evidence.

It is important to consider that the validation of indicators took place in a period in which Mexico and the entire world were in a pandemic due to SARS-CoV-2. The pandemic conditioned, on the one hand, the low demand for services due to confinement measures, the difficulties in transportation, or the fear of getting infected; and on the other hand, the decrease in the supply of health services. The health services that were the most affected by this situation were family planning, prenatal, obstetric, and postnatal care, newborn care, child care, adolescents, sexual, and reproductive health, chronic diseases, and nutrition programs. The latter were completely interrupted in some cases (24). As a result of this situation, an increase of up to 10% in acute malnutrition in the Latin American and Caribbean region has been estimated as a direct consequence of the decrease in maternal and child health care services (24, 25).

The principal limitation identified during the validation process was the inability to measure the feasibility of all indicators due to the lack of clinical records. Clinical records are the source documents for most studies on the health care process; however, it is important to be aware of the deficiencies that prevail in these records in clinical practice in general. Some authors point out that the lack of adequate clinical records is not incompatible with good clinical practice (21). Nevertheless, if the quality criteria evaluated in these records were sufficiently relevant for the adequate care of a certain health problem, this situation could be considered a factor of poor quality of care.

In the pilot study, neglect or even lack of clinical records was observed; this did not allow the evaluation of relevant aspects of the care process for the different forms of malnutrition. This lack of clinical records may be due to an omission of care activities carried out by health personnel, which could cause problems in monitoring the health status of health service users. The absence of clinical records is also evidence of the quality of the care process.

In addition, for some indicators, the number of medical records necessary to be able to estimate the reliability statistics was not completed. However, it was considered necessary to maintain some indicators that presented difficulties for their measurement, due to the relevance indicated by the scientific literature and the experts. For example, regarding the indicators of the preconception stage such as “Folic acid supplementation,” no records were found about patients who were planning a pregnancy; therefore, to assess the care provided to this group, alternative sources must be used, such as the registry of pregnant patients. Even though feasibility and reliability could not be evaluated in the indicators belonging to preconception, we decided to keep them in the group of the 16 final indicators due to the importance of the nutritional status at this stage in the course and outcome of pregnancy in both the mother and the offspring.

Despite the limitations, to our knowledge, this is the first effort to design and validate a systematic set of indicators to evaluate the quality of maternal and child nutritional care. We consider it relevant to have indicators that allow evaluating the quality of maternal and child nutritional care that make it possible to highlight areas of opportunity, variability of care, and progress in the improvement of care. This study provides a path to focus on quality-improvement initiatives within PHC. The 16 indicators developed were used to assess the quality of maternal and child nutritional care in health units of PHC in six states in Mexico. The results of this evaluation will be published shortly.

## Conclusion

The systematic use of the 16 indicators at the PHC to monitor and evaluate the quality of maternal and child nutritional care could contribute substantially to improving the nutritional status during preconception, pregnancy, postpartum, infancy, and preschool stage at the individual and population level.

## Data availability statement

The original contributions presented in this study are included in the article/Supplementary material, further inquiries can be directed to the corresponding author.

## Author contributions

MA-M, SH, IO-G, and SB-M: conceptualization and design. MA-M, IO-G, SH, AB-M, OA-R, MV-C, EM, CP, SB-M, MK-H, CM-M, MM, BS, MB, MS, and FC: investigation. MA-M, IO-G, AB-M, OA-R, CP, MV-C, MB, MS, AC, BS, and FC: supervision of the investigation. MA-M, IO-G, SH, AB-M, OA-R, MV-C, EM, MK-H, CP, SB-M, MB, MS, and FC: methodology. IO-G, SB-M, AC, BS, and AT-D: data curation and organization of the databases. EM, AB-M, and OA-R: formal statistical analysis. MA-M, IO-G, AB-M, OA-R, MK-H, CM-M, MM, and FC: review and edition of the manuscript. All authors contributed to manuscript revision, read, and approved the submitted version.

## References

[1] Chakhtoura N Chinn JJ Grantz KL Eisenberg E Artis Dickerson S Lamar C Importance of research in reducing maternal morbidity and mortality rates. *Am J Obstet Gynecol.* (2019) 221:179–82. 10.1016/j.ajog.2019.05.050 31492377 PMC7586732

[2] Murray CJL Aravkin AY Zheng P Abbafati C Abbas KM Abbasi-Kangevari M Global burden of 87 risk factors in 204 countries and territories, 1990–2019: a systematic analysis for the Global Burden of Disease Study 2019. *Lancet.* (2020) 396:1223–49. 33069327 10.1016/S0140-6736(20)30752-2 PMC7566194

[3] Shamah-Levy T Romero-Martínez M Barrientos-Gutiérrez T Cuevas-Nasu L Bautista-Arredondo S Colchero MA *Encuesta Nacional de Salud y Nutrición 2020 sobre Covid-19. Resultados Nacionales.* Cuernavaca: Instituto Nacional de Salud Pública (2021). 10.21149/12580 34098602

[4] Marchi J Berg M Dencker A Olander EK Begley C. Risks associated with obesity in pregnancy, for the mother and baby: a systematic review of reviews. *Obes Rev.* (2015) 16:621–38. 10.1111/obr.12288 26016557

[5] Martorell R Zongrone A. Intergenerational influences on child growth and undernutrition. *Paediatr Perinat Epidemiol.* (2012) 26 (Suppl. 1):302–14. 10.1111/j.1365-3016.2012.01298.x 22742617

[6] World Health Organization [WHO], United Nations Children’s Fund [UNICEF]. *A Vision For Primary Health Care in The 21st Century: Towards Universal Health Coverage and The Sustainable Development Goals.* Geneva: World Health Organization (2018).

[7] World Health Organization [WHO]. *Essential Nutrition Actions: Mainstreaming Nutrition Through The Life-Course.* Geneva: World Health Organization (2019).

[8] UNICEF. *Nutrition, for Every Child: UNICEF Nutrition Strategy 2020–2030.* New York, NY: UNICEF N Y U N Child Fund (2020).

[9] World Health Organization [WHO]. *Essential Nutrition Actions: Improving Maternal, Newborn, Infant and Young Child Health and Nutrition.* Geneva: World Health Organization (2013). 25473713

[10] Scott N Delport D Hainsworth S Pearson R Morgan C Huang S Ending malnutrition in all its forms requires scaling up proven nutrition interventions and much more: a 129-country analysis. *BMC Med.* (2020) 18:356. 10.1186/s12916-020-01786-5 33183301 PMC7661178

[11] Ruel MT Alderman H Maternal and Child Nutrition Study Group. Nutrition-sensitive interventions and programmes: how can they help to accelerate progress in improving maternal and child nutrition? *Lancet.* (2013) 382:536–51. 10.1016/S0140-6736(13)60843-0 23746780

[12] Tunçalp Ö Were WM MacLennan C Oladapo OT Gülmezoglu AM Bahl R Quality of care for pregnant women and newborns-the WHO vision. *BJOG.* (2015) 122:1045–9. 10.1111/1471-0528.13451 25929823 PMC5029576

[13] Lorini C Porchia BR Pieralli F Bonaccorsi G. Process, structural, and outcome quality indicators of nutritional care in nursing homes: a systematic review. *BMC Health Serv Res.* (2018) 18:43. 10.1186/s12913-018-2828-0 29373962 PMC5787252

[14] Moick S Simon J Hiesmayr M. Nutrition care quality indicators in hospitals and nursing homes: a systematic literature review and critical appraisal of current evidence. *Clin Nutr.* (2020) 39:1667–80. 10.1016/j.clnu.2019.07.025 31447247

[15] Billah SM Saha KK Khan ANS Chowdhury AH Garnett SP Arifeen SE Quality of nutrition services in primary health care facilities: implications for integrating nutrition into the health system in Bangladesh. *PLoS One.* (2017) 12:e0178121. 10.1371/journal.pone.0178121 28542530 PMC5436890

[16] Brenes-Monge A Yáñez-Álvarez I Meneses-León J Poblano-Verástegui O Vértiz-Ramírez JJ Saturno-Hernández PJ. Approach to the quality of care during pregnancy, delivery and postpartum in women withobstetric risk factors in Mexico. *Salud Publica Mex.* (2020) 62:798–809. 10.21149/11974 33620976

[17] Brouwers MC Kho ME Browman GP Burgers JS Cluzeau F Feder G Development of the AGREE II, part 1: performance, usefulness and areas for improvement. *CMAJ.* (2010) 182:1045–52. 10.1503/cmaj.091714 20513780 PMC2900328

[18] Saturno-Hernández PJ. *Métodos y Herramientas Para la Monitorización de la Calidad En Servicios de Salud.* Cuernavaca: Instituto Nacional de Salud Pública (2015).

[19] Secretaría de Salud. *Formatos e Instructivos de Unidades Médicas 2019.* Sistema de Información en Salud (SIS) (2019). Available online at: http://www.dgis.salud.gob.mx/contenidos/sis/formatos2022.html (accessed March 10, 2022).

[20] Lassi ZS Kedzior SGE Tariq W Jadoon Y Das JK Bhutta ZA. Effects of preconception care and periconception interventions on maternal nutritional status and birth outcomes in low- and middle-income countries: a systematic review. *Nutrients.* (2020) 12:606. 10.3390/nu12030606 32110886 PMC7146400

[21] Donabedian A. The quality of care. How can it be assessed? *JAMA.* (1988) 260:1743–48. 10.1001/jama.260.12.1743 3045356

[22] Kötter T Blozik E Scherer M. Methods for the guideline-based development of quality indicators–a systematic review. *Implement Sci.* (2012) 7:21. 10.1186/1748-5908-7-21 22436067 PMC3368783

[23] Stelfox HT Straus SE. Measuring quality of care: considering measurement frameworks and needs assessment to guide quality indicator development. *J Clin Epidemiol.* (2013) 66:1320–7. 10.1016/j.jclinepi.2013.05.018 24018344

[24] Villalobos Dintrans P Maddaleno M Granizo Román Y Valenzuela Delpiano P Castro A Vance C Interrupción de servicios de salud para embarazadas, recién nacidos, niños y niñas, adolescentes y mujeres durante la pandemia de COVID-19: proyecto ISLAC 2020. *Rev Panam Salud Pública.* (2021) 45:e140. 10.26633/RPSP.2021.140 34737772 PMC8559667

[25] Roberton T Carter ED Chou VB Stegmuller AR Jackson BD Tam Y Early estimates of the indirect effects of the COVID-19 pandemic on maternal and child mortality in low-income and middle-income countries: a modelling study. *Lancet Glob Health.* (2020) 8:e901–8. 10.1016/S2214-109X(20)30229-1 32405459 PMC7217645

