# Supplementary material for: Development and validation of a new set of indicators to assess the quality of maternal and child nutritional care at the primary care
Source: Front Med (Lausanne). 2022 Dec 7;9:1011940. doi: 10.3389/fmed.2022.1011940 (PMC9769120; doi:10.3389/fmed.2022.1011940)
Supplement: Supplementary file 1 [file Table_1.pdf]

Supplementary Material

Supplementary Table 1. Search logarithms in Pubmed by life stage

**Preconception**	(“preconception period” OR preconception) AND ("Preconception Care"[Mesh] OR "Nutrition Assessment"[Mesh] OR "Nutrition Therapy"[Mesh] OR "prevention and control" [Subheading] OR "Health Promotion"[Mesh]) AND ("Malnutrition"[Mesh] OR "Body Weight"[Mesh] OR "Anemia"[Mesh] OR "Deficiency Diseases"[Mesh] OR "Nutrition Disorders"[Mesh] OR "Nutritional Physiological Phenomena"[Mesh])
**Pregnancy**	("Pregnancy"[Mesh] OR "Pregnancy Trimester, First"[Mesh] OR "Pregnancy Trimester, Second"[Mesh] OR "Pregnancy Trimester, Third"[Mesh]) AND ("Prenatal Care"[Mesh] OR "Nutrition Assessment"[Mesh] OR "Nutrition Therapy"[Mesh] OR "prevention and control" [Subheading] OR "Health Promotion"[Mesh]) AND ("Malnutrition"[Mesh] OR "Body Weight"[Mesh] OR "Anemia"[Mesh] OR "Deficiency Diseases"[Mesh] OR "Nutrition Disorders"[Mesh] OR "Gestational Weight Gain"[Mesh] OR "Fetal Growth Retardation"[Mesh] OR "Nutritional Physiological Phenomena"[Mesh])
**Postpartum**	("Postpartum Period"[Mesh] OR “postpartum” OR "Lactation"[Mesh]) AND ("Postnatal Care"[Mesh] OR "Nutrition Assessment"[Mesh] OR "Nutrition Therapy"[Mesh] OR "prevention and control" [Subheading] OR "Health Promotion"[Mesh]) AND ("Malnutrition"[Mesh] OR "Body Weight"[Mesh] OR "Anemia"[Mesh] OR "Deficiency Diseases"[Mesh] OR "Nutrition Disorders"[Mesh] OR "Nutritional Physiological Phenomena"[Mesh])
**Infancy**	("infant"[MeSH Terms] OR "Infant, Newborn"[Mesh]) AND ("Nutrition Assessment"[Mesh] OR "Nutrition Therapy"[Mesh] OR "prevention and control" [Subheading] OR "Health Promotion"[Mesh]) AND ("Infant Nutrition Disorders"[Mesh] OR "Malnutrition"[Mesh] OR "Body Weight"[Mesh] OR "Anemia"[Mesh] OR "Deficiency Diseases"[Mesh] OR "Nutritional Physiological Phenomena"[Mesh]) NOT ("Nutritional Support"[Mesh])
**Preschool age**	("Child, Preschool"[Mesh]) AND ("Nutrition Assessment"[Mesh] OR "Nutrition Therapy"[Mesh] OR "prevention and control" [Subheading] OR "Health Promotion"[Mesh]) AND ("Child Nutrition Disorders"[Mesh] OR "Body Weight"[Mesh] OR "Growth Disorders"[Mesh] OR "Deficiency Diseases"[Mesh] OR "Nutritional Physiological Phenomena"[Mesh]) NOT ("Nutritional Support"[Mesh])
