# Supplementary material for: Development and validation of a new set of indicators to assess the quality of maternal and child nutritional care at the primary care
Source: Front Med (Lausanne). 2022 Dec 7;9:1011940. doi: 10.3389/fmed.2022.1011940 (PMC9769120; doi:10.3389/fmed.2022.1011940)

Supplementary Material

Supplementary Figure 1. Flowchart of the development of the indicators to evaluate the quality of maternal and child nutritional care in PHC

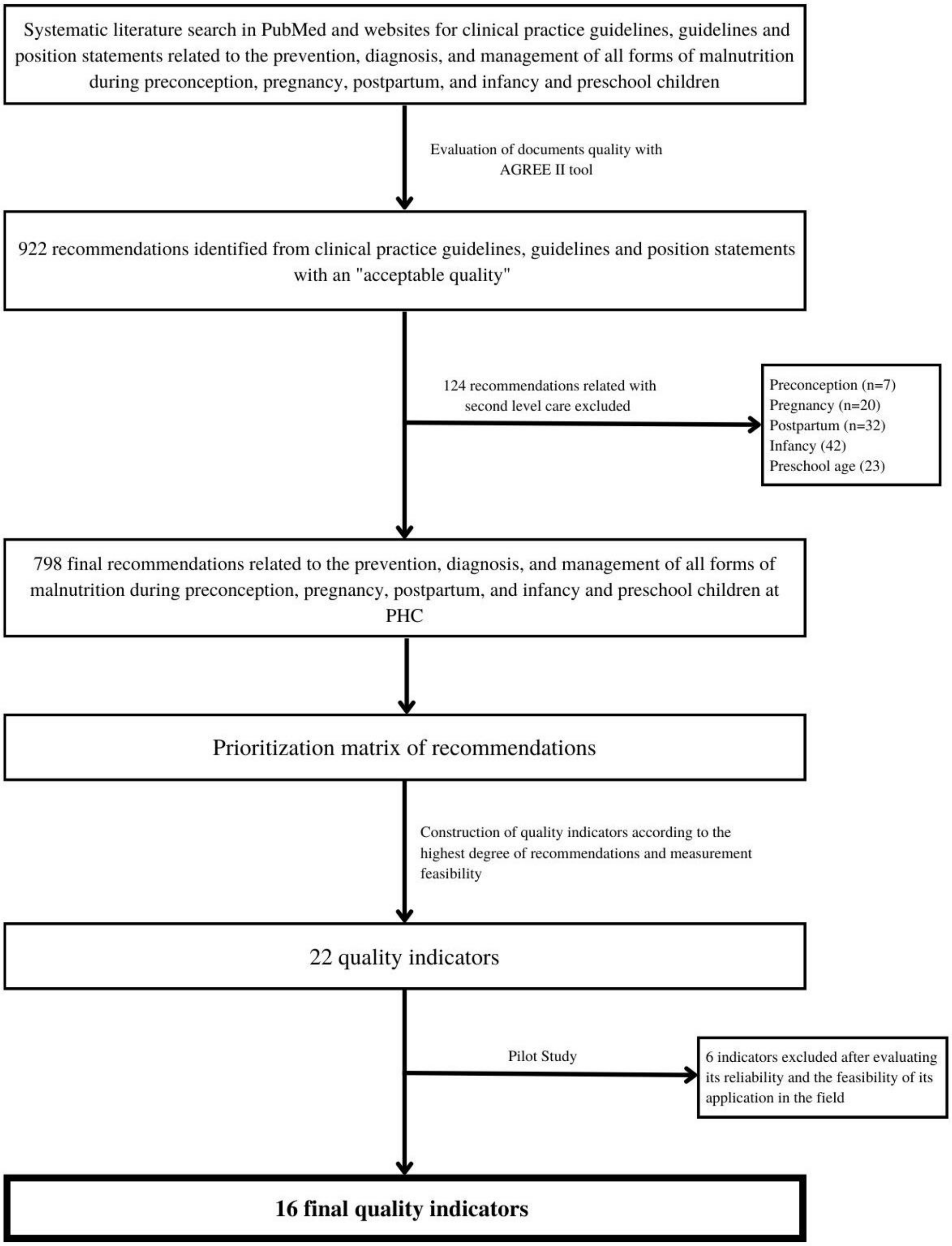

Supplement: Supplementary file 2 [file Image_1.pdf]
